# Future publishing in the environmental sector

**DOI:** 10.1007/s44274-023-00002-4

**Published:** 2023-05-30

**Authors:** Charis M. Galanakis

**Affiliations:** 1 Research and Innovation Department, Galanakis Laboratories, Chania, Greece; 2 Taif University, Taif, Saudi Arabia; 3 Food Waste Recovery Group, ISEKI Food Association, Vienna, Austria

The environmental sector faces numerous technical and economic challenges that reflect clean processing, legislation issues, and societal changes. This fact has affected the entire sector significantly, forcing public authorities and involved companies to focus on developing products, services, and processes that address people's demand for a cleaner environment and a more sustainable world. Indeed, there is an extensive dialogue about the need of the environmental industry to introduce innovations, propose strategic and greener development options, optimize resource reuse, and ultimately become sustainable. However, even though researchers continuously work in this direction, the demonstration and communication of their innovative products, services, and treatment technologies meet several obstacles and significant restrictions within the publishing industry. Along this line, a new scientific reference is needed to publish all the recent trends and emerging issues in the environmental science and technology field.

## Consecutive crises have emerged the need for environmental protection, sustainable development, and a shift to a climate-neutral economy

Humanity's highly carbon-intensive development model for over two centuries has led to dire repercussions for the environment and is continuously depleting food, water, material, and energy resources [1, 2]. In addition, over the last few years, we have witnessed severe changes in societal norms due to a condensed period of consecutive crises. Starting from the increased events related to climate change impacts, moving to the pandemic outbreak and the Russian–Ukrainian war, our societies are struggling to recover at many levels [3]. For example, climate change has affected the origins and transmission routes and the virulence and persistence of bacteria, fungi, food pathogens, parasites, marine and freshwater algal blooms, and vectors pathogenic to animals and plants [4]. Furthermore, the war has not only caused an extreme humanitarian crisis, but it has severe detrimental consequences, e.g., water availability is reduced, the properties of soils are impacted, ecosystem services are damaged, air quality is diminished, and leakage of radiation is possible [5, 6].

Following these examples, the emergence of the above crises uncovered our development model's weaknesses in securing stable ecosystems and protecting citizens from pollutants [7]. There is a substantial scientific consensus today that humans significantly cause major global environmental problems and climate change. Therefore, modern societies should support retrofitting developed environments to make them more sustainable. Moreover, this consensus led to urgent political acts worldwide targeting adopting a climate-neutral economy and a more sustainable growth model.

## Recent crises increased pressure on the scientific publishing industry to accelerate the review process and increase accessibility

Research is primarily communicated through peer-reviewed articles, although other forms as book chapters, conference proceedings, webinars, videos, and social media posts, are also becoming increasingly popular. After the pandemic outbreak, a new trend in the publishing industry was observed. In particular, the urgent need for information regarding the prevention of SARS-CoV-2 spread, the development of treatment methods, and the suggestion of strategies for societal re-arrangements led the scientific community to "fast-track" and open publishing via social media posts and scientific blogs. This practice continues nowadays due to the emergence of new crises (e.g., climate change, geopolitical conflicts, etc.), having a significant disadvantage: the lack of the necessary peer-review process by experts in the field. Nevertheless, the noted gap in the communication of scientific knowledge is not big enough to restrict authors from publishing since the number of researchers is increasing exponentially. Besides, submitting a paper to an electronic journal and waiting for a semester to get a response belongs to the previous century. The millennial researchers cannot wait that long: they demand fast and accurate decision-making and choices. This tendency should not worry the scientific community as a sketchy approach as it reflects an evolution of the publishing industry based on improved and realistic journal handling practices [8].

Today, one month from submission to decision is a realistic timeframe for all journals, e.g., a couple of days for administration tasks and a few weeks for the review process. Accessibility is also crucial; thus, switching to OA options is unavoidable in the new era of publishing. However, article processing charges for OA publication should be reasonable for as many researchers worldwide as possible.

## Future publishing with Discover Environment

Given the increasing threats from multiple hazards, shifting towards more sustainable systems will be critical for the planet's survival. Ensuring the environment provides a safe, livable, and vibrant place will be vital for human well-being, and sustainable development is essential to ensure environmental degradation and destruction are avoided. Along this line, researchers are more focused on the quality of ecosystems (broadly covering both agricultural and urban contexts), targeting environmental change and a sustainable growth model that respects the environment and the planet's boundaries. The interest in this topic will remain relevant for the foreseeable future. However, the debates will soon shift to ensuring the application of the most efficient solutions and advancements. Environmental science, technology, and engineering help improve the natural environment, keep the air clean, and provide pure water and land.

*Discover Environment,* as a transdisciplinary and open-access journal, aspires to fill in the respective gap in the scientific literature. The journal provides a leading platform for rapidly disseminating knowledge and advances in environmental science, chemistry, technology, and engineering. It publishes innovative and high-impact original research papers, viewpoints, review articles, case studies, and short communications presenting basic, methodological, and applied research and innovation across the full range of disciplines concerned with the environment. The journal particularly welcomes papers highlighting food waste valorization approaches, biorefinery efforts for generating biobased products, transformative resilience pathways towards bioeconomy, and studies dealing with the new policy trends of the circular and climate-neutral economy. Focus will also be given to investigations that fall into the scope of the United Nations Sustainable Development Goals (SDGs) and particularly those addressing SDG 6 (clean water and sanitation), SDG7 (affordable and clean energy), SDG13 (climate action), SDG14 (life below water), and SDG15 (life on land).

*Discover Environment* topics include ecology, biodiversity, biotic and abiotic stresses, environmental toxicology and human health, conservation, and restoration of degraded ecosystems (atmospheric, agriculture, marine, forestry, etc.), renewable energy, climate change, nature-based solutions, and biomass valorization. The food, water, and energy nexus are of particular interest to the journal, together with environmental soil science and land contamination, hydrology and geochemistry modeling, water supply, security, sustainable growth, urban environmental problems, carbon footprint, life cycle assessment, and cleaner production. Managing water and wastewater, solid, hazardous, and industrial waste, emissions, and air pollution are also covered. Finally, future publishing in the environmental sector requires innovation strategies, policies, economics, industrial ecology, sustainability, and eco-design practices, as well as integrating data science and industry 4.0 applications.

## Data Availability

All data used in this study is publicly available in databases (Scopus, Web of Science, ScienceDirect, etc.).
